# Stance and weight distribution after tibial plateau leveling osteotomy in fore limb and hind limb amputee dogs

**DOI:** 10.1186/s12917-020-02402-7

**Published:** 2020-06-10

**Authors:** Ron Ben-Amotz, David Dycus, David Levine, Andréia Gonçalves Arruda, Nicholas Fagan, Denis Marcellin-Little

**Affiliations:** 1 Small Animal Orthopedics, Koret School of Veterinary Medicine, Rehovot, Israel; 2 The Hebrew University of Jerusalem, P.O Box 12, 76100 Rehovot, Israel; 3 American College of Veterinary Surgeons - Small Animal, Veterinary Orthopedic and Sports Medicine Group, 10975 Guilford Rd Annapolis Junction, Philadelphia, MD 20701 USA; 4 Department of Physical Therapy, The University of Tennessee at Chattanooga, 615 McCallie Ave Dept #3253, Chattanooga, USA; 5 A194 Department of Veterinary Preventive Medicine, College of Veterinary Medicine, The Ohio State University, Columbus, USA; 6 Veterinary Surgery and Emergency Center, 1114, Philadelphia, South Front Street 19147 USA; 7 American College of Veterinary Surgeons, Philadelphia, USA; 8 American College of Veterinary Sports Medicine and Rehabilitation (Charter), Philadelphia, USA; 9 Small Animal Orthopedic Surgery, University of California, Davis, USA

**Keywords:** Dog, Amputation, Tibial plateau leveling, TPLO, Weight distribution

## Abstract

**Background:**

Little is known about the weight distribution to the remaining limbs for amputee dogs that undergo orthopedic surgery.

The objective of the paper was to describe stance and weight distribution after tibial plateau leveling osteotomy (TPLO) in forelimb and in hind limb amputees (Amp_TPLO_) and to compare them to four-legged TPLO patients (4L_TPLO_) and amputees without TPLO (Amp). Weight bearing distribution at a stance was compared between groups. Joint angles of forelimb and hind limb joints in a sagittal plane, hind limb orientation in a frontal plane, and pelvic orientation in a transverse plane (pelvic tilt) were measured and compared between groups.

**Results:**

Joint angles, hind limb abduction, and pelvic tilt of Amp_TPLO_ and Amp did not differ statistically. Mean weight bearing in the operated hind limb was higher for Amp_TPLO_ than 4L_TPLO_. Mean weight bearing for thoracic limbs of Amp_TPLO_ and 4L_TPLO_ did not differ statistically. Weight bearing of the hind limb of Amp_TPLO_ and Amp did not differ statistically.

**Conclusions:**

The position of the center of mass and posture of Amp_TPLO_ and Amp does not differ. The weight distribution and posture of Amp is not impacted negatively by TPLO.

## Background

Amputation of a limb is performed to manage complex fractures, neoplasia, osteomyelitis, soft tissue infection, and limb deformities that cause severe disability in dogs [1]. While recovery after thoracic limb or hind limb amputation is satisfactory in most instances the posture and mobility of amputee dogs vary widely [1–3]. The loss of a limb results in an increase in the ground reaction forces and contact times of the remaining limbs. These increases in ground reaction forces and contact times are greater after the loss of a forelimb than a hind limb [4, 5]. Hind limb amputee dogs also adjust to the loss of a hind limb by increasing tarsal joint range of motion as well as increase range of the cervicothoracic and thoracolumbar vertebral regions [6]. A significant dilemma whether an amputee dog that develops a major orthopedic disease such as CCL rupture, can have an acceptable limb function following an orthopedic repair. Rupture of the cranial cruciate ligament (CCL) and the subsequent development of progressive osteoarthritis is the most common cause of hind limb lameness in dogs. Osteotomy procedures such as the tibial plateau leveling osteotomy (TPLO), are commonly recommended [7].

Objective clinical information related to recovery after TPLO is lacking. To the authors’ knowledge, only one scientific article describes patient outcomes and owner satisfaction for dogs undergoing orthopedic surgery after an amputation [8]. In that article, 11 amputees had a CCL rupture and nine of those underwent TPLO. The survey respondents were satisfied with outcome of amputee dogs that had a TPLO surgery. However, information describing stance and the position of the center of mass in amputees after surgical stabilization following CCL rupture has not been reported in the literature.

The objectives of the current study were to measure the weight distribution and posture of forelimb and hind limb amputee dogs after a TPLO (Amp_TPLO_), to compare those to weight distribution and posture in amputee dogs not undergoing a TPLO (Amp) and to four-legged dogs after a TPLO (4L_TPLO_). We hypothesized that Amp_TPLO_ dogs would bear more weight on their remaining hind limb than 4L_TPLO_ and would bear less weight on their operated leg than Amp. We also hypothesized that Amp_TPLO_ would have more hind limb abduction and pelvic tilt than 4L_TPLO_.

statistical significance was *P* < 0.05.

## Results

Twenty-eight dogs were enrolled in the study: seven Amp_TPLO_, 10 Amp, and 11 4L_TPLO_. Among Amp, eight dogs had a hind limb amputation and two dogs had a thoracic limb amputation. For AMP_TPLO_, four dogs had a thoracic limb amputation, and three dogs had a hind limb amputation. For 4L_TPLO_ three TPLOs were performed on the left limb and eight in the right limb. Detailed information for dogs included in this case series is presented on Table 1. The mean age for Amp_TPLO_ at the time of surgery was 7.0 years (range, 1.5 to 12.6 years) and mean weight was 19.1 ± 12.9 kg. Four males (57%) and three females (43%) were included. The mean age for Amp was 9.8 years (range, 2.0 to 13.1 years) and mean weight was 21.8 ± 6.7 kg. Seven females (70%) and three males (30%) were included. The mean age for the 4L_TPLO_ was 5.6 years (range, 3.3 to 8.9 years) and mean weight was 22.9 ± 10.8 kg. Six females (54%) and five males (45%) were included. Dog breeds are reported in Table 1.

**Table 1:** Demographic data for 7 amputees who underwent a TPLO (Amp_TPLO_), 10 control amputees (Amp), and 11 control four-legged dogs (4L_TPLO_) who underwent a TPLO

Group	Age (years)	Weight (kg)	Sex	Breed	Amputated limb	Side of TPLO
Amp_TPLO_	12.6	6.5	F	Chihuahua mix	RF	R
Amp_TPLO_	8.0	8.6	M	Pomeranian mix	RR	L
Amp_TPLO_	1.3	5.0	F	Miniature poodle	LF	L
Amp_TPLO_	6.0	30.1	M	Pitbull	RR	R
Amp_TPLO_	7.0	40.0	M	German SD	LF	R
Amp_TPLO_	9.9	21.4	M	Mix	LR	R
Amp_TPLO_	4.0	22.7	F	German SD	RF	R
Amp	10.1	10.3	F	American Eskimo	RR	–
Amp	13.1	22.1	F	Pointer	RR	–
Amp	10.8	21.5	F	Pitbull	RR	–
Amp	10.3	17.1	F	Mix	LR	–
Amp	11.4	30.4	M	Golden retriever	RR	–
Amp	9.0	22.3	F	Mix	RR	–
Amp	18.0	20.0	F	German SP	RF	–
Amp	2.0	34.0	F	Husky mix	LR	–
Amp	9.0	24.0	M	Bull terrier	RR	–
Amp	4.0	17.0	M	Beagle	LF	–
4L_TPLO_	4.8	22.7	F	Am Staffordshire	–	R
4L_TPLO_	3.3	28.1	M	Labrador retriever	–	R
4L_TPLO_	7.0	13.1	M	Mix	–	R
4L_TPLO_	3.4	32.3	M	Labrador retriever	–	L
4L_TPLO_	4.4	41.9	F	Great Pyrenees	–	L
4L_TPLO_	5.5	7.7	F	Poodle x pug mix	–	R
4L_TPLO_	6.0	36.4	F	Bouvier des Flandres	–	R
4L_TPLO_	7.9	14.0	M	Cocker spaniel	–	R
4L_TPLO_	8.0	21.3	M	Pitbull	–	L
4L_TPLO_	2.6	19.2	F	Shepherd mix	–	R
4L_TPLO_	8.9	17.3	F	Mix	–	R

Abbreviations: *SD* Shepherd dog; *SP* Shorthaired pointer; *Am* Amercian; *RF* Right forelimb; *LF* Left forelimb; *RR* Right hind limb; *LR* Left hind limb; *R* Right; *L* Left

The weight distribution among Amp_TPLO_, Amp, and 4L_TPLO_ dogs did not differ statistically (*P* = 0.56, 0.50, and 0.94, respectively for comparison among groups).

**Fig. 1:**
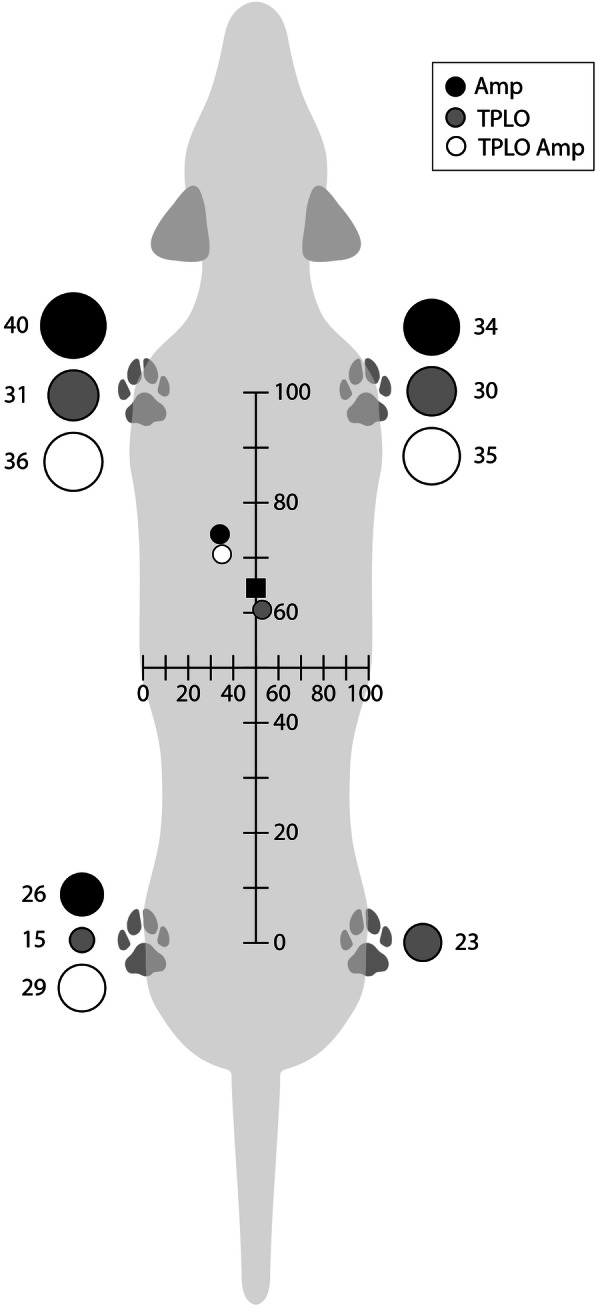
Mean percentage loads resisted by each limb and resulting center of mass position for amputee dogs (*N* = 10, black circles), control TPLO (*N* = 11, grey circles), and TPLO amputees (*N* = 7, white circles). The size of the circles is proportional to the loads resisted by each limb. The position of the center of mass for normal quadruped dogs is shown as a black square.

Joint angles of the thoracic limbs including shoulder, elbow, and carpus in Amp_TPLO_ and Amp did not differ statistically (107.6° ± 10.2° and 99.9° ± 13.3°, *P* = 0.60; 123.3° ± 13.9° and 125.0° ± 13.4°, *P* = 0.91; and 130.55° ± 13.83°, 135.30° ± 16.29°, *P* = 0.73, respectively). Similarly, hind limb joint angle measurements including hip, stifle and tarsal angles in Amp_TPLO_ and Amp did not differ statistically (114.8° ± 13.5° and 110.4° ± 13.1°, *P* = 0.49; 154.7° ± 8.7° and 145.7° ± 10.9°, *P* = 0.42; and 210.5° ± 6.7° and 217.9° ± 11.6°, *P* = 0.82, respectively). There was no statistical difference between pelvic tilt and limb adduction among Amp_TPLO_ and Amp (*P* = 0.56 and 0.45, respectively). The mean ± SD percentage weight bearing in the operated limb was higher in Amp_TPLO_ (27.1 ± 3.3%, range, 23.0 to 32.1%) than 4L_TPLO_ (15.5 ± 2.1%, range, 11.9 to 18.8%, *P* < 0.001). The percentage weight distribution in the hind limb for Amp_TPLO_ (29.1 ± 2.8%, range 26.5 to 32.1%) and Amp (26.4 ± 3.4%, range 21.3 to 32.0%) did not differ (*P* = 0.22); (Fig. 1). The mean percentage weight bearing in thoracic limbs did not differ between Amp_TPLO_ (61.4 ± 4.1%) and 4L_TPLO_ (57.1 ± 13.1%, *P* = 0.62).

## Discussion

The purpose of this study was to identify and report objective data pertaining to amputee dogs with CCL injury that have undergone a TPLO procedure. The decision to proceed with surgical stabilization of the affected stifle in these animals may be challenging for both clinicians and owners, particularly when an osteotomy procedure is recommended. The authors believe that the perception of a challenging recovery and undesirable outcome may result in many of these cases remaining untreated. Therefore, the data presented in this paper has the potential to assist both clinicians and owners in the decision-making process. The objectives of the current study were to describe stance and weight distribution in Amp_TPLO_ and to compare posture (joint angle, pelvic tilt, limb varus), weight distribution, and weight shift, between Amp_TPLO_, and two other groups of dogs: Amp and 4L_TPLO_. We rejected the hypothesis that the posture of Amp_TPLO_ differed from Amp. The lack of postural differences between Amp_TPLO_ and Amp may inform the decisions of owners and clinicians considering TPLO to manage CCL injury in an amputee. We rejected the hypothesis that Amp_TPLO_ would shift more weight to their thoracic limbs compared to Amp as there was no difference in weight distribution to the thoracic limbs between the two groups. Amp_TPLO_ appear to re-establish appropriate thoracic limb weight bearing over a relatively short period of time. When comparing Amp_TPLO_ and Amp, the similar position of the center of mass position, confirms the fact that posture is not impacted by TPLO in amputees. We rejected the hypothesis that AMP_TPLO_ would carry less weight on their operated limbs compared to Amp. These results suggest a positive response to TPLO on the remaining limb.

Kinetic and kinematic gait analysis has been shown to be more objective than visual examination in the detection of lameness and in evaluating limb function [9, 10]. Kinetic and kinematic studies have been completed to give objective data to pet owners as to how dogs will adapt following an amputation [6, 11, 12]. Kinetic changes after amputation indicate that amputee dogs use a compensatory mechanism that involves the unaffected diagonal limb pair [6, 12].

We selected a weight distribution platform in the current study since amputee patients may not be able to reliably ambulate at the required velocity to obtain valid trials on force plate or pressure sensitive walkways. In addition, weight distribution platforms are less costly than force plate and pressures sensitive walkways, require less space, and require fewer skills during data acquisition [13–15]. We accepted the hypothesis that AMP_TPLO_ carry more weight on their operated limb compared to 4L_TPLO_. In a recent article describing the long-term outcome of 4L_TPLO_ using force plate analysis, symmetry indices of the TPLO group did not differ from a normal control group 6 to 12 months after surgery [16].

When evaluating the effects of limb amputation on standing weight distribution in the remaining three limbs, dogs with a previous hind limb amputation had the largest mean increase in weight bearing in the contralateral thoracic limb [14]. Interestingly, our results suggest that Amp_TPLO_ increase the weight placed on their ipsilateral thoracic limb. In contrast to four-legged control dogs which bear 60% of their weight in the forelimbs and 40% in the hind limbs [11]. After a hind limb amputation, dogs bear 74% of their weight on their thoracic limbs and 26% in the remaining hind limb [5]. In the current study, Amp_TPLO_ appear to behave similarly to those Amp with regards to thoracic limb weight distribution.

When facing a cranial cruciate CCL ligament injury in Amp, a TPLO appears to be an appropriate treatment option because changes in posture or weight distribution after surgery are not expected. Proactive monitoring of orthopedic disease in the front limbs may be advisable in dogs with a previous limb amputation that undergo a surgical repair such as a TPLO. In addition, when determining candidacy for repair of the remaining hind limb, disease of the contralateral forelimb should be thoroughly evaluated.

This study had limitations: The procedure was performed by several surgeons. Data before TPLO were not available, the small sample size could have prevented enough power to detect potential statistical associations, the variability among forelimb and hind limb amputee patients within the Amp_TPLO_ and Amp. Both forelimb and hind limb amputees that had undergone a TPLO had acceptable weight bearing on their operated limbs with minimal postural difference compared to Amp. Since the current study was limited to an evaluation of stance, it is unclear how the gait at a walk and trot of forelimb and hind limb amputees that underwent a TPLO were impacted by the combination of an amputation and a TPLO. The gait adaptations of forelimb and hind limb amputees at trot have been described [6, 12] and are complex. Briefly, in forelimb amputees, the ipsilateral hind limb assume the role of a forelimb and a hind limb [12]. In pelvic limb amputees, changes affect mainly the contralateral tarsus and the cervico-thoracic and lumbosacral vertebral regions [6]. Further work should be performed to determine what long-term changes occur with weight bearing in amputee dogs undergoing a TPLO. Joint angles were measured by placing dots on anatomic landmarks and measuring angles between lines formed by connecting these dots. The method used had good repeatability in a previous study [17]. However, its accuracy is not known. The relatively small differences in joint angles among groups appeared to be proportional to the small difference in position of the center of mass, indirectly suggesting that the measurements were accurate. Although kinetic and kinematic analysis provide more objective data regarding weight bearing in dogs compared to visual observation, stance analysis may be a more readily available modality and may be easier to use.

## Conclusion

In conclusion, a TPLO procedure in amputees results in an acceptable weight bearing with minimal postural difference compared to Amp.

## Methods

### Dog sample

All dogs undergo either a forequarter amputation or a coxofemoral disarticulation at least 8 weeks or more prior to being enrolled in the study. Patients treated at three veterinary specialty practices^1^^,^^2^^,^^3^ for complete CCL tear during the study period were eligible for inclusion if they were Amp_TPLO_, Amp, or 4L_TPLO_, if owners signed an informed consent, and if a follow-up 8 weeks or more after TPLO was performed. No meniscal injuries were identified during surgery. TPLO were stabilized with a bone plate and a combination of locking and non-locking screws.^4^ Craniocaudal and mediolateral stifle radiographs were acquired before surgery, immediately after surgery, and 8 weeks or more after surgery. Dogs were excluded from the study if an orthopedic disease problem other than CCL injury was detected during the orthopedic evaluation.

### Data collected

Data collected included breed, sex, age at the time of surgery, limb amputated, presenting complaint, physical examination and orthopedic findings, the presence or absence of pre-existing joint disease, complications, and outcome.

**Fig. 2:**
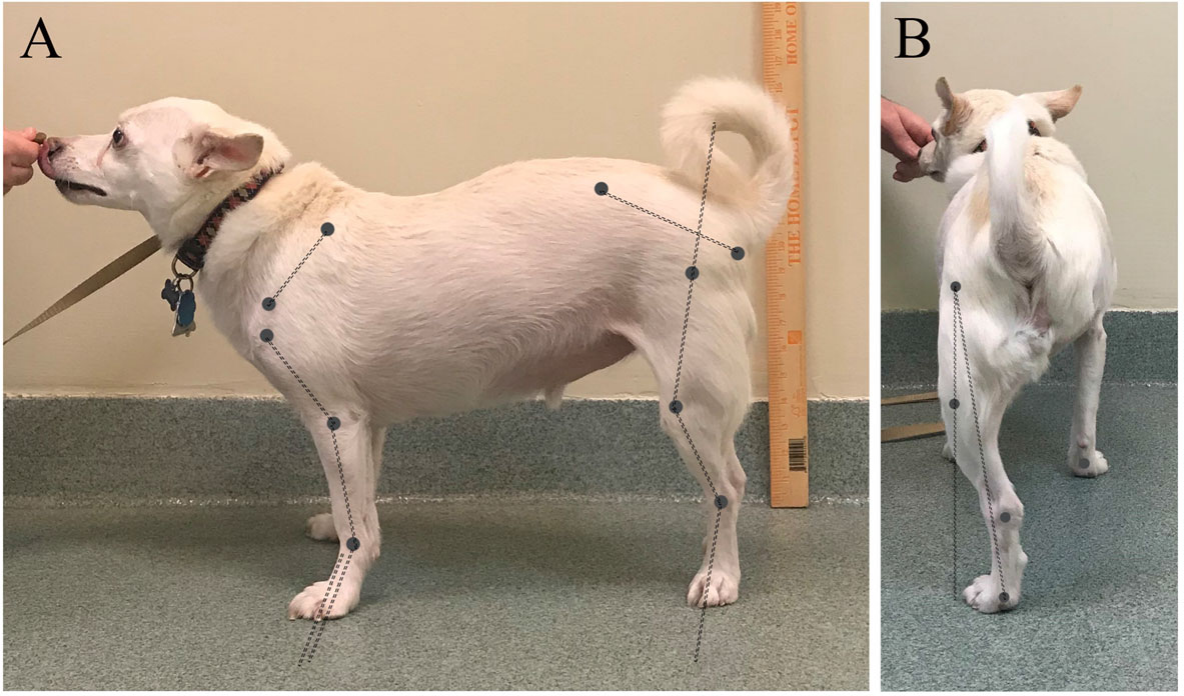
Pictures acquired from the side (**a**) and back (**b**) of an amputee dog who underwent a TPLO 8 weeks earlier. Joints angle are measured by tracing specific anatomic landmarks and drawing lines along or joining those landmarks [18]. For the dog in the picture the shoulder was held at 104.1°, the elbow at 153.1°, the carpus at 213.6°, the hip at 105.1°, the stifle at 146.4°, and the tarsus at 145.1°. Pelvic limb abduction, measured as the angle formed by a vertical line and a line joining the center of the hip joint and metatarsal pad, was 9.1°

Standing weight distribution was measured using a weight distribution platform.^5^ Dogs stood on the platform naturally, with their head and neck facing forward. For each patient, fifteen (on and off the platform) measurements of weight distribution were recorded and means were calculated. Photographs of the left and right sides, back and front of each dog were acquired using a camera placed at the level of stifle or elbow joint (Fig. 2). The camera^6^ was placed 2 m from the dog and was perpendicular to the long axis of the dog. For dogs with long hair, the hair was clipped to aid in identification of anatomic landmarks.

Shoulder, elbow, carpus, hip, stifle, and tarsus angles were measured in a sagittal plane, hind limb alignment in a frontal plane (abduction or adduction), and pelvic orientation in a transverse plane (pelvic tilt) were measured using a previously established protocol [18] and image analysis software.^7^ The intersection of a line parallel to the spine of the scapula and the line joining the craniocaudal midpoint at the proximal aspect of the humerus and the lateral humeral epicondyle formed the shoulder joint angle. The intersection of the line joining the craniocaudal midpoint at the proximal aspect of the humerus and the lateral humeral epicondyle and the line joining the lateral humeral epicondyle and the craniocaudal midpoint at the distal aspect of the antebrachium formed the elbow joint. The intersection of the line joining the lateral humeral epicondyle and the craniocaudal midpoint at the distal aspect of the antebrachium and a line parallel to the dorsal aspect of the third metacarpal bone formed the carpus. The intersection of the line joining the tuberischiadicum and sacrale and the line joining the greater trochanter and the craniocaudal midpoint at the distal aspect of the femur formed the hip joint angle. The intersection of the line joining the greater trochanter and the craniocaudal midpoint at the distal aspect of the femur and the line joining the craniocaudal midpoint of the proximal portion of the tibia and the lateral malleolus formed the stifle joint. The intersection of the line joining craniocaudal midpoint of the proximal portion of the tibia and the lateral malleolus and a line parallel to the dorsal aspect of the third metatarsal bone formed the tarsus. Hind limb adduction was the angle formed by a line joining the greater trochanter and the center of the metatarsal pad and a vertical line. Left-sided pelvic tilt was the angle formed by a line joining the left and right tuber ischiadicum and a horizontal line. Positive left-sided pelvic tilt meant that the left tuber ischiadicum was lower than the right tuber ischiadicum.

### Statistical analysis

Sample size calculation was based on historic data in dogs with CCL injury [19] and was planned with the intent to detect a differences in mean weight bearing (peak vertical force / body weight) between Amp_TPLO_ and 4L_TPLO_ on the limb that underwent a TPLO at an α of 0.5 and a power (β) of 0.8. Sample size calculation^8^ yield a sample size of 3 to statistically detect a difference of 20% between groups and a sample size of 9 to statistically detect a difference of 10% between groups. Statistical analyses were conducted using statistical analysis software.^9^ Due to the small sample size and violation of assumptions of normality and equality of variance, nonparametric tests were used for comparison of variables of interest between groups, including Wilcoxon rank sum tests (Mann-Whitney tests) for comparisons of median values and Spearman’s rank correlation coefficients for correlation analyses. Wilcoxon rank sum tests were used to compare joint angles, pelvic tilt, and limb varus for AMP_TPLO_ and Amp. Since only two amputee dogs were missing a thoracic limb, joint angles were compared only in animals missing a pelvic limb. Correlation analysis was conducted separately for Amp_TPLO_ and Amp, in regards to percentage of weight bearing in the thoracic limbs, and each of the joint angles for shoulder, elbow and carpus. In dogs missing a hind limb, we tested whether AMP_TPLO_ were shifting more weight forward on the side opposite their amputation compared to Amp. For these analyses, side to side data were flipped so that the missing limb was the right hind limb for all Amp and Amp_TPLO_. Data were also flipped so that all 4L_TPLO_ had TPLO of the left hind limb. We tested whether AMP_TPLO_ placed more weight on their operated limb compared to 4L_TPLO_ and whether AMP_TPLO_ placed more weight on their hind limb compared to Amp. For all analyses, statistical significance was *P* < 0.05.

## Data Availability

Additional data would be to offer the spreadsheet that was used for the analyses. It would be the spreadsheet that the statistician used. Raw data with no identifiable information could be available upon reasonable request to the corresponding author.

## Abbreviations

Amp: Amputees
Amp_TPLO_: Amputees undergoing TPLO
CCL: Cranial cruciate ligament
4L_TPLO_: Four-legged dogs undergoing TPLO
TPLO: Tibial plateau leveling osteotomy

## Footnotes

1 Veterinary Specialty and Emergency Clinic, Philadelphia, PA

2 MedVet Carmel, Carmel, IN

3 Sunstone Veterinary Specialists, Portland, OR

4 DePuy Synthes TPLO, West Chester, PA

5 Stance analyzer, Companion Animal Health, Newark, DE

6 12 M-pixel front-facing camera, iPhone 7S, Apple, Cupertino, CA

7 Adobe Creative Suite, Adobe Systems, San Jose, CA

8 https://www.stat.ubc.ca/~rollin/stats/ssize/n2.html, accessed, January 25, 2020

9 STATA/ IC 14.2, StataCorp LP, College Station, TX
